# Correlation in the Coronal Angle between Knee and Hindfoot Was Observed in Patients with Rheumatoid Arthritis Unless Talocrural Joint Was Destroyed

**DOI:** 10.1155/2017/4051706

**Published:** 2017-10-23

**Authors:** Kohei Nishitani, Hiromu Ito, Yoshiharu Shimozono, Moritoshi Furu, Masayuki Azukizawa, Motomu Hashimoto, Masao Tanaka, Tsuneyo Mimori, Shuichi Matsuda

**Affiliations:** ^1^Department of Orthopaedic Surgery, Kyoto University Graduate School of Medicine, Kyoto, Japan; ^2^Department of the Advanced Medicine for Rheumatic Diseases, Kyoto University Graduate School of Medicine, Kyoto, Japan; ^3^Department of Clinical Immunology and Rheumatology, Kyoto University Graduate School of Medicine, Kyoto, Japan

## Abstract

The purpose of this study is to investigate the compensatory correlation between knee and hindfoot in patients with rheumatoid arthritis (RA). This cross-sectional study included 218 patients (407 lower extremities). Radiographs of the hindfoot and full-length posteroanterior hip-to-calcaneus standing radiographs were evaluated. The destruction of the hindfoot was evaluated using the Larsen grading system. The coronal angular deformity of the knee and hindfoot was evaluated by the femorotibial angle (FTA) and the angle between the tibial shaft and the entire hindfoot (tibiohindfoot angle, THFA). The correlation between FTA and THFA was determined by Pearson's coefficient. For all patients, FTA correlated to THFA (*R* = 0.28, *p* < 0.001). The correlation was observed as long as the talocrural joint was preserved (Larsen grade ≤ 2), even if the subtalar joint had been destroyed (Larsen grade ≥ 3). However, the correlation was not observed when the talocrural joint was destroyed (Larsen grade ≥ 3, *R* = −0.02, *p* = 0.94). The pain in the hindfoot did not correlate with FTA or THFA. In conclusion, a compensatory deformity of the hindfoot against the deformity of the knee was observed in RA, and the correlation was lost when talocrural joint was destroyed.

## 1. Introduction

In the coronal alignment of the lower limb, patients with osteoarthritis (OA) exhibit a collaborative correlation of the angle of the knee and the hindfoot [1–4]. Varus knee deformity of the OA knee tends to have valgus hindfoot, and vice versa. In some previous reports, the change in the alignment of the knee due to a surgical intervention, such as total knee arthroplasty (TKA) [2, 3, 5] or high tibial osteotomy, [6] affects the alignment of the hindfoot within a short time after the surgery. Meanwhile, in rheumatoid arthritis (RA), it is not evident whether the compensatory correlation of the angle of the knee and hindfoot which is observed in OA patients can be applied using the same theory.

RA is a common autoimmune disease which is characterized by many features, primarily synovial inflammation and joint destruction which leads to disability for the patient [7]. Theoretically, all of the joints can be involved, but the knee joint is one of the most commonly affected and also the joint most frequently requiring surgery [8]. The hindfoot is also often affected in patients with RA, and the prevalence of the subtalar joint is reported as 29% to 32% [9, 10]. In terms of the coronal deformity, the valgus deformity of the hindfoot is reported in 25% of the RA patients [11]. In a recent ultrasonic study, synovitis occurred more frequently compared with the radiographic change in hindfoot and the prevalence of talocrural, subtalar, and talonavicular synovitis is 76%, 71%, and 59%, respectively [12]. Radiographically, the talocrural joint is less affected in patients with RA and the change in the subtalar joint proceeds to the talocrural joint in many cases [13].

Nakada et al. report a significant correlation between femorotibial and tibiocalcaneal angles (TCA) in patients with RA [14]. In their evaluations of the radiographs, the TCA was used as the coronal hindfoot alignment, but the total hindfoot (talus-calcaneus) was not evaluated, and they used the different radiographs to evaluate the FTA and TCA. To this end, we used the hip-to-calcaneus (H-to-C) radiographs [15] to evaluate the entire lower limb from hip joint to calcaneus in one radiogram and to evaluate the total hindfoot from the proximal talus to the distal calcaneus in one radiogram.

We hypothesized that the hindfoot compensated for the malalignment of the knee in patients with RA, but only when the hindfoot was preserved. We aimed to distinguish between the talocrural and subtalar joints to investigate the compensatory alignment change of the hindfoot. We also evaluated the relationship between the pain of the patients and the alignment of the hindfoot.

## 2. Methods

### 2.1. Patients

A total of 370 patients who visited the rheumatoid arthritis center of our university hospital between March 2012 and December 2012 were eligible to participate in this cross-sectional study. The study was approved by the Ethics Committee of our university, and all subjects provided written informed consent. All patients met the criteria for the 2010 American College of Radiology/the European League Against Rheumatism classification criteria for RA [16]. Of the 740 lower limbs of the 370 patients, 122 limbs (58 right, 64 left) were excluded because of previous surgery; 99 (49 right, 50 left) were excluded because of the lack of full-length radiographs of the lower extremity; 88 (43 right, 45 left) were excluded because of the lack of pain score data; and 24 (12 right, 12 left) were excluded because of the lack of a Larsen radiographic grade for the talocrural and/or subtalar joints. Thereafter, a total of 407 lower limbs (208 right, 199 left) from 218 patients met the criteria for evaluation in this study. The demographic data for the 218 patients is summarized in Table 1.

### 2.2. Radiographs and Measurements

**Figure 1:**
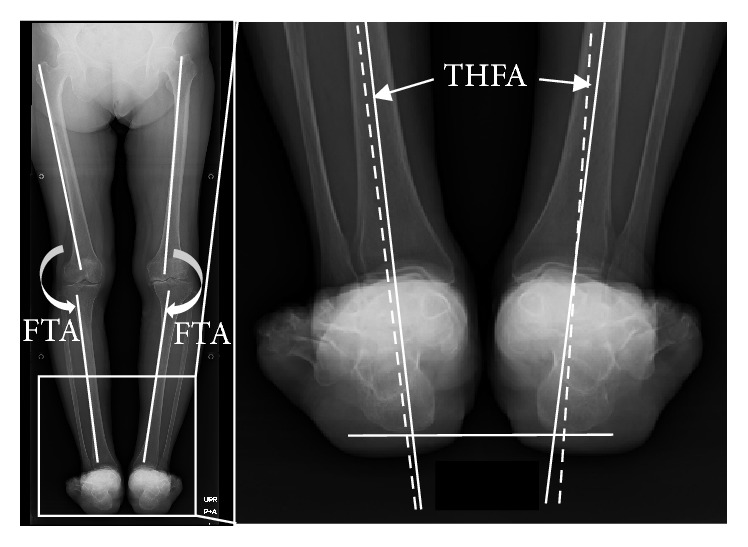
The hip-to-calcaneus (H-to-C) radiograph is shown in the left and the FTA was determined as shown. In the right radiograph, there is an enlargement of the left radiograph, the THFA, which is the internal angle of the tibial axis (solid white line). The dotted white line between the center of the talar dome and the lowest point of the calcaneus was measured.

Full-length posteroanterior H-to-C radiographs of the lower extremities were taken as previously described by Haraguchi et al. [15]. Briefly, the patients stood on the radiolucent platform facing the X-ray cassette with their forefoot dorsi-flexion on the pads. The distance between the cassette and the X-ray tube was set to 2 meters, and the center of the knee joint was focused using 85 kV voltage, 200 mA current, and 0.45-second exposure time. For the alignment of the knee, the femorotibial angle (FTA), defined as the external angle between the femoral shaft axis and the tibial shaft axis, was measured. For the alignment of the hindfoot, the tibiohindfoot angle (THFA), which was a modification of the Saltzman hindfoot angle [1, 17], was defined as the internal angle between the tibial shaft axis and the coronal hindfoot axis, which was the line connecting the center of the talar dome and the lowest point of the calcaneus (Figure 1). These measurements were made by board-certified orthopaedic surgeons of the Japanese Orthopaedic Association. To express the FTA, varus knee indicates larger FTA and valgus knee indicates smaller FTA. With regard to the THFA, a valgus hindfoot has a large THFA (plus value) and a varus hindfoot has a small THFA (minus value). Anteroposterior and lateral weight bearing radiographs of the hindfoot were also taken to give a Larsen radiographic grade from 0 to 5, where 0 is no destruction and 5 is mutilating change [18]. These evaluations were performed by two certified rheumatologists of the Japan College of Rheumatology. Talo-1st metatarsal angle (Talo-1st MTA), which is the angle between long axis of the talus and long axis of the first metatarsal and calcaneal pitch, which is a line drawn along the lower border of the calcaneus and line between the inferior border of the calcaneus and the inferior aspect of the medial sesamoid bone. Valgus, neutral, and varus alignments of the hindfoot were defined as THFA ≤ −2.1, −2 ≤ THFA ≤ 4.4, and 4.5 ≤ THFA, respectively. This classification is based on the previous analysis of 100 THFAs in patients with RA who had no deformity or destruction in any joints in either of the lower legs (as defined as being within mean ± 1SD of neutral alignment of the hindfoot, data not shown). A Larsen grade of less than 2 for both the talocrural and subtalar joints of the hindfoot was defined as the hindfoot preserved, and a Larsen grade of more than 3 for either the talocrural or subtalar joint was defined as hindfoot affected (Table 2).

### 2.3. Pain in the Hindfoot

The part of general pain (maximum score is 40 points) in ankle/hindfoot scale of the Japanese Society for Surgery of the foot ankle-hindfoot (JSSF ankle/hindfoot scale) (maximum score is 100 points) was used for the evaluation of the pain in the hindfoot in this cohort [19, 20]. In this scale, scores were recorded as follows: 40 points = no pain; 30 points = mild pain (sometimes painful while in motion); 20 points = moderate pain (always painful while in motion); and 0 points = severe pain (always painful).

### 2.4. Statistics

Pearson's correlation coefficients were used to determine the correlation between the FTA and THFA. Spearman's correlation coefficients were used to determine the correlation between radiographic measurement and pain score. The strength of the correlation was defined as follows: 0–0.19 = no correlation; 0.20–0.39 = weak correlation; 0.40–0.69 = moderate correlation; and 0.70–1.00 = strong correlation. The pain scores among the 3 hindfoot alignment groups (valgus, neutral, and varus) were analyzed with either a one-way or two-way ANOVA, as appropriate, and a Tukey post hoc test. *p* values of less than 0.05 were considered statistically significant.

## 3. Results

**Figure 2:**
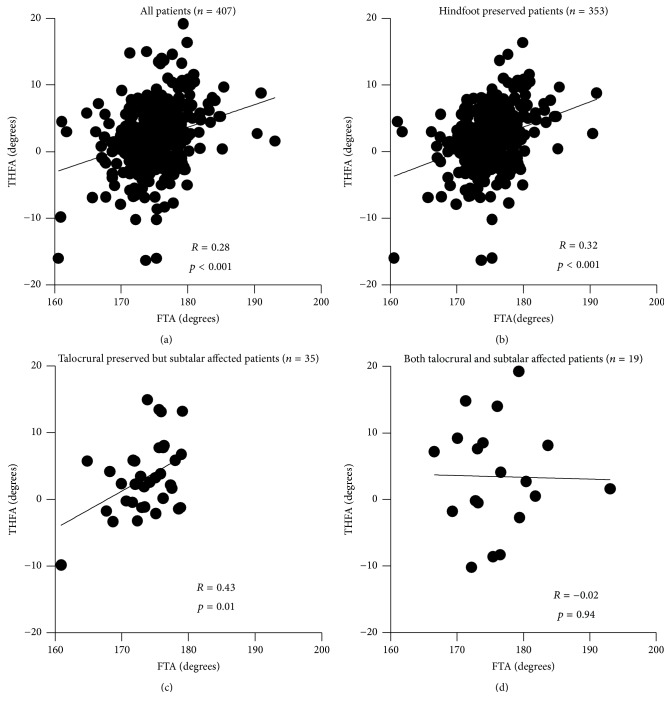
The correlation between FTA and THFA of (a) all patients in this cohort, (b) hindfoot preserved patients (Larsen grade of both talocrural subtalar joints < 2), (c) talocrural joint preserved (Larsen grade < 2) but subtalar joint affected (Larsen grade > 3), and (d) both talocrural and subtalar joints affected (Larsen grade > 3) is shown. Note that there is significant correlation between FTA and THFA as long as talocrural joint is preserved.

**Figure 3:**
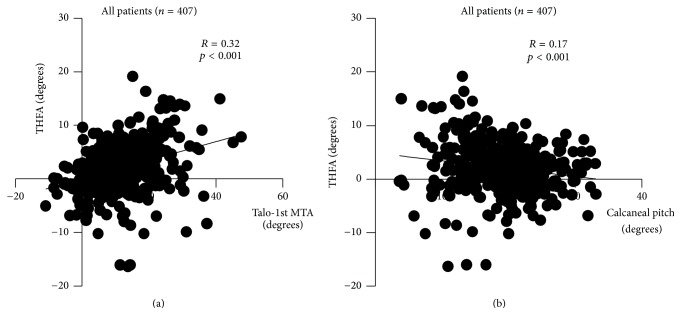
The correlation between Talo-1st MTA and THFA of (a) and the correlation between calcaneal pitch and THFA (b) are shown.

THFA correlated with FTA only when the talocrural joint was preserved. Though the correlation was not strong, THFA was significantly correlated with FTA (*R* = 0.28, *p* < 0.001) in the analysis of the all legs in the cohort (*n* = 407), regardless of deformity of the hindfoot (Figure 2(a)). The condition of the talocrural and subtalar joint of the patients categorized by Larsen grade was shown in Table 2. In the legs in which the talocrural and subtalar joint were preserved (Larsen grade = 0, 1, 2) (*n* = 353), there was a positive correlation between TCA and FTA (*R* = 0.32, *p* < 0.001) (Figure 2(b)). Even when the subtalar joint was in an advanced stage of RA (Larsen grade = 3, 4, 5), the THFA was correlated with the FTA (*R* = 0.43, *p* = 0.01), unless the talocrural joint was destroyed (*n* = 35) (Figure 2(c)). However, when both the talocrural and subtalar joints were affected (*n* = 19), THFA no longer correlated with FTA (*R* = −0.02, *p* = 0.94) (Figure 2(d)). There was no patient whose talocrural joint was destroyed (Larsen grade = 3, 4, 5) but subtalar joint was preserved (Larsen grade = 0, 1, 2). The sagittal alignment of the forefoot also affected the coronal alignment of the hindfoot. Talo-1st MTA weakly correlated to the THFA (*R* = 0.32, *p* < 0.001) (Figure 3). On the other hand, there was very weak correlation between calcaneal pitch and THFA (*R* = 0.17, *p* < 0.001), which we did not think it was clinically meaningful correlation (Figure 3).

**Figure 4:**
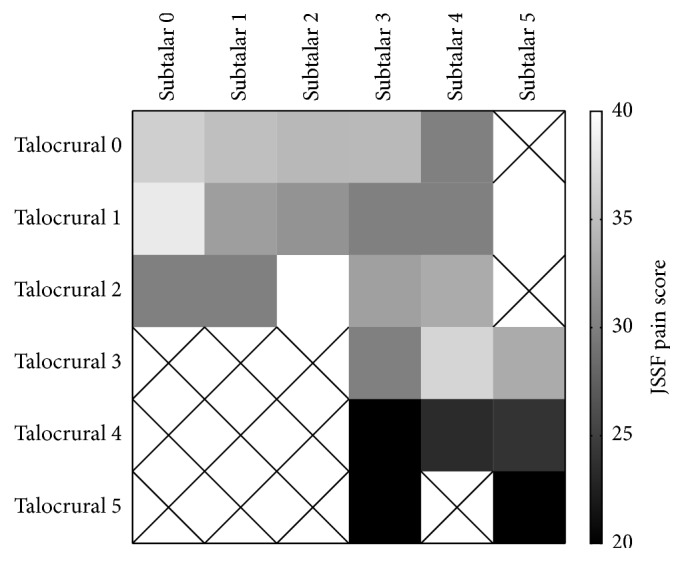
Heatmap of the JSSF pain scores of all patients, categorized by the Larsen grade of the talocrural and subtalar joints. Boxes marked with “X” indicate no patients in the category.

Pain in the hindfoot was analyzed using the Larsen grades of the talocrural and subtalar joints. A heatmap (Figure 4) indicates a lower JSSF pain score around the right bottom corner, indicating that patients with the worst talocrural and subtalar Larsen grades suffered severe pain. JSSF pain scores were weakly correlated with the Larsen grades for subtalar joint pain (*R* = −0.27, *p* < 0.001) and did not correlate with the Larsen grades for talocrural joint pain (*R* = −0.18, *p* < 0.001).

The hindfoot or knee alignment did not affect the pain in the hindfoot in patients with RA. There was no correlation between the JSSF pain score and the FTA (*R* = −0.06, *p* = 0.21) or THFA (*R* = −0.08, *p* = 0.13). Using the THFA, the 407 legs were classified into 3 alignments: valgus (*n* = 110), neutral (*n* = 236), and varus (*n* = 61). There was no significant difference in JSSF pain scale scores among these 3 alignments (*p* = 0.44) (Table 3). Hindfoot affected patients showed lower JSSF pain score than hindfoot preserved patients (*p* < 0.001) (Table 3). Interestingly, the JSSF pain score for patients with a neutral alignment was significantly lower (worse) for patients with an affected hindfoot compared with patients with a preserved hindfoot (*p* = 0.016). This trend was not conspicuous for the varus or valgus alignments (Table 3).

## 4. Discussion

In patients with RA in this study, a weak but significant correlation between the alignment of the knee and the hindfoot was observed, as with the correlation of OA patients. Interestingly, a significant correlation was observed unless the talocrural joint was destroyed. However, there was no correlation when the talocrural joint was affected in patients with RA. The result of this correlation is that even when the subtalar joint was severely affected in patients with RA, the hindfoot appeared to be able to compensate for the alignment abnormality of the knee, as long as the talocrural joint was preserved. The importance of the talocrural joint for the alignment of the lower limb was identified in this study.

The potential of the correlation of the ankle or hindfoot might be weaker in RA patients compared with others. In patients with OA of the knee, the moderate correlation between knee and ankle alignment is reported [1, 21]. For patients of RA with low Larsen grades, the correlation in the previous study [14] and in this cohort were both weak. The object of OA study was patients undergoing TKA, who tend to have severe deformity, and the deformity of the knee was milder in RA cohorts. The correlation is likely to increase as the deformity of the knee progress [1], and this may be one of the reasons that the correlation of the RA cohorts including this study was weak compared to the studies of OA.

In the pioneer study of Nakada et al. [14], the TCA does not correlate to the FTA when the Larsen grade of the subtalar joint is 4 or more. The main difference in our study is the method of radiography; we used H-to-C methods [15] in order to measure FTA and THFA in one X-ray film. Also, the information from the talar dome to the bottom of calcaneus was included in order to know the whole coronal axis of the hindfoot. If the coronal deformity of the hindfoot is analyzed using the calcaneal axis, the deformity is led mostly by subtalar joint. However, in this study, the information for the entire hindfoot could be evaluated by using the H-to-C methods. Norton et al. report that 72% of the variance of the hindfoot is explained by the change of the subtalar joint [1], which means that the remaining 28% should be influenced by other components, including the talocrural joint. Furthermore, we investigated almost twice as many limbs (407 limbs) compared with Nakada et al. (205 limbs) so that a significant correlation could be determined. Although Nakada et al. determine similar correlation coefficients, they might not able to establish statistical significance.

Clearly, there was no patients who had affected talocrural but preserved subtalar joint. Subtalar joint is more susceptible in RA than talocrural joint [13], and only about 15% of the RA patients who had more than 20-year disease duration show affected talocrural joint (Larsen grade ≥ 3) [22]. The destruction of the subtalar joint seemed to proceed the destruction of the talocrural joint, judging from the distribution of the patients in Figure 4 in this study. The loss of the function of the subtalar joint might cause the excessive mechanical stress to the talocrural join which might cause the further destruction of the talocrural joint. Meanwhile, coronal alignment of the hindfoot was also correlated to one parameter in sagittal alignment. Matsumoto et al. report that flat foot is a characteristic deformity from midfoot involvement, with the change of talo-1st metatarsal angle [22]. Taken together, this suggested that the adjacent joint such as talonavicular or calcaneocuboid joint in midfoot may influence the coronal alignment of the hindfoot.

This study also focused on the difference in pain associated with the deformity of the hindfoot, although the pain of the hindfoot was not different among the 3 alignment groups. In this study, the JSSF pain score increased as the destruction of the hindfoot. For patients with a valgus alignment of the hindfoot, there was no significant difference in pain score based on the severity of the destruction of the joints in patients with RA. A significant difference in pain was observed for patients with a neutral alignment, not in valgus alignment. Moreover, the difference of the mean pain score between the hindfoot preserve and hindfoot affected patients became larger as the THFA alignment became varus from valgus. Even in the destruction in patients with RA in Larsen grade, the hindfoot which had the capacity to compensate the alignment change might be the less affected hindfoot, when the whole structure including such as soft tissue was considered. Takenaka et al. reported that limbs with valgus alignment of the hindfoot compensate following TKA; however, limbs with varus alignment of the hindfoot did not compensate following TKA [2]. Thus, valgus alignment of the hindfoot might maintain higher function with less pain. However, valgus deformity of the hindfoot is one of the frequent deformities in the patients of RA regardless of the deformity of the knee. The valgus hindfoot could be result from either the deformity of the hindfoot itself or the compensation deformity against the deformity of the knee, and further study is needed to investigate the cause of the alignment change and relation between the alignment and pain of the hindfoot.

We have described new insights into the compensatory deformity of the hindfoot for the knee joint deformity, but this study is not free from limitations. First, the static condition of the knee and hindfoot angle was evaluated in this study, but dynamic knee and hindfoot laxity or instability were not taken into consideration. Second, the coronal deformity of the knee and ankle joints was only evaluated by H-to-C X-ray, so three-dimensional information was not available, and the effect of the femoral and tibial rotation could not be excluded. In addition, the FTA of H-to-C X-ray may not be identical to the FTA in the standard full-length anteroposterior X-ray which is used in other studies [1, 14], since knee joint might be slightly flexed because of the dorsiflexion or forefoot. Third, the pain in the hindfoot was described, but this pain was only as reported by the patient, so the localization of the pain was not included. Fourth, the involvement of the soft tissue was not taken into account. Synovitis, hydroarthritis, or the condition of the tendon or the ligament seemed to affect more dynamic instability, but there is a possibility that these parameters had a small influence in this study. Fifth, this was a cross-sectional study and the longitudinal change of the relationship between the deformity of the knee and the hindfoot was not investigated. In the study with OA knee, the ankle promptly adapts to the postsurgical change in the angle of the knee because the hindfoot is mostly normal [3, 5]. No evaluation was performed to determine if the same ability of adaptation was present in the patients with RA or to determine the occurrence of temporary or permanent pain in RA after TKA.

## 5. Conclusions

In this study, the correlation between the knee and hindfoot angle was confirmed in patients with RA. However, the preservation of the talocrural joint was needed for the compensatory deformity of the hindfoot. A further longitudinal study is warranted to determine the levels of pain and overall clinical condition at follow-up and the effect of the correction of the malalignment of the knee on the ankle and hindfoot after TKA.

## Figures and Tables

**Table 1:** Demographic data.

	Median (min–max)
	or number (%)
Age, years	62.0 (29–92)

Disease duration, years	13.9 (0–50)

Stage	I; 60, II; 107, III; 64, IV; 176

DAS28-ESR	3.12 (0.084–6.59)

Anti-CCP positivity, *n* (%)	183 (84%)

RF positivity, *n* (%)	178 (82%)

HAQ	0.709 (0–2.875)

bDMARDs (*n*)	69 (32%)

Steroid, *n* (%)	80 (37%)
Amount of steroid, mg	4.3 (1.0–10.0)

MTX, *n* (%)	158 (72%)
Amount of MTX, mg	7.3 (2.0–20.0)

VAS = visual analogue scale; DAS28 = Disease Activity Score 28; ESR = erythrocyte sedimentation rate; ACPA = anti-cyclic citrullinated peptide antibody; RF = rheumatoid factor; HAQ = health assessment questionnaire; bDMARDs = biological disease-modifying antirheumatic drugs; MTX = methotrexate.

**Table 2:** Patients categorized by Larsen grade of talocrural and subtalar joint.

		Subtalar joint	
		Preserved	Affected
		Larsen grade (0, 1, 2)	Larsen grade (3, 4, 5)
		*N*	*N*
Talocrural joint	Preserved	353	35
	Larsen grade (0, 1, 2)		
	Affected	0	19
	Larsen grade (3, 4, 5)		

*N* = number.

**Table 3:** JSSF pain scores categorized by the condition of the hindfoot and THFA alignment.

	THFA alignment		
	Valgus	Neutral	Varus
All patients	33.7 ± 8.1	34.6 ± 7.9	35.4 ± 8.0
(*n* = 407)	(*n* = 110)	(*n* = 236)	(*n* = 61)

Hindfoot preserved	34.3 ± 8.1	35.2 ± 7.6	36.0 ± 7.7
(*n* = 353)	(*n* = 90)	(*n* = 210)	(*n* = 53)

Hindfoot affected	31.0 ± 7.8	30.4 ± 9.2^*∗*^	30.0 ± 9.3
(*n* = 54)	(*n* = 20)	(*n* = 26)	(*n* = 8)

Significant difference was confirmed in the Row factor (hindfoot preserved versus hindfoot affected) in two-way ANOVA (*p* < 0.001). Data is expressed as mean ± SD. ^*∗*^*p* < 0.05 by Turkey's post hoc test compared with patients with preserved hindfoot in the neutral alignment.
